# Poly(Adenosine Diphosphate Ribose) Polymerase (PARP) Inhibitors in the Treatment of Advanced Ovarian Cancer: A Narrative Review

**DOI:** 10.7759/cureus.68463

**Published:** 2024-09-02

**Authors:** Deepika Dewani, Arpita Jaiswal, Pravin Karwade

**Affiliations:** 1 Obstetrics and Gynaecology, Jawaharlal Nehru Medical College, Datta Meghe Institute of Higher Education and Research, Wardha, IND

**Keywords:** rucaparib, niraparib, olaparib, brca mutations, homologous recombination deficiency (hrd), dna repair mechanisms, advanced ovarian cancer, parp inhibitors

## Abstract

Poly(adenosine diphosphate ribose) polymerase (PARP) inhibitors have appeared as a revolutionary approach to treating advanced ovarian cancer, particularly in patients with breast cancer (BRCA) mutations and homologous recombination deficiency (HRD). This narrative review explores PARP inhibitors' clinical efficiency, safety, and changing role in this context. PARP inhibitors, such as olaparib, niraparib, or rucaparib, provide considerable benefits regarding progression-free survival expansion and overall outcomes improvement in first-line maintenance and recurrent settings. The underlying mechanisms, patient selection criteria, and resistance patterns are discussed, alongside insights into combination therapies to overcome resistance and enhance therapeutic efficacy. Ongoing clinical trials and future potential for personalized therapy approaches using PARP inhibitors for advanced ovarian cancer are also highlighted. However, despite these drugs' phenomenal ability to revolutionize treatment protocols for such cancer types, several challenges remain: toxicity management, cost, and development of resistance will require more research to optimize their use or broaden patient populations who can benefit from them.

## Introduction and background

Ovarian cancer ranks among the most fatal malignancies affecting women, typically recognized at later stages as it remains foreshadowed by no observable indications during its early stages (the first two or three phases). Though there have been recent advancements in surgery and chemotherapy, the prognosis for advanced ovarian cancer is still grim due to the high recurrence rate, coupled with resistance towards conventional treatments. However, poly(adenosine diphosphate ribose) polymerase (PARP) inhibitors have changed the game altogether for individuals with advanced ovarian cancer, especially those carrying breast cancer (BRCA1/2) mutations or so-called homologous recombination deficiencies (HRDs) [1]. The DNA repair pathways are targeted by PARP inhibitors to exploit the cancer cells’ synthetic lethality due to their homologous recombination repair (HRR) deficiency. These agents effectively kill HRD-positive cancer cells by inhibiting PARP enzymes necessary to repair single-strand DNA breaks. Therefore, it is on record that olaparib, niraparib, and rucaparib have clinically proven successful in improving progression-free survival rates among patients suffering from advanced ovarian cancer [2]. Besides treating BRCA-mutated tumours, even more, advanced forms of therapy utilizing PARP inhibitors have been reported to be effective against a greater number of patients who have HRD [3]. In addition, many ongoing trials explore this combination with other modalities, such as manipulations, to name a few, aimed at developing novel ways of using medicamentos antitumorales and reducing toxicity.

## Review

Search methodology

To conduct a narrative review on the role of PARP inhibitors in the treatment of advanced ovarian cancer, a narrative search methodology was employed. Relevant literature was identified through a structured search strategy in significant databases, including PubMed, Scopus, and Web of Science, using keywords and phrases such as “PARP inhibitors,” “advanced ovarian cancer,” and “treatment outcomes.” The search was limited to articles published in the last decade to ensure the inclusion of recent advancements and clinical data. Inclusion criteria encompassed peer-reviewed studies, clinical trials, and review articles that discussed the efficacy, mechanisms, and side effects of PARP inhibitors in ovarian cancer. Data were extracted from selected studies, focusing on treatment protocols, patient responses, and clinical outcomes. The quality of evidence was assessed using established criteria, and findings were synthesized to provide a comprehensive overview of the current state of knowledge and future directions for research in this therapeutic area.

Mechanism of action of PARP inhibitors

DNA Repair Pathways

Overview of homologous recombination (HR) and its role in DNA repair: HR is a critical DNA repair mechanism that ensures the accurate repair of double-strand breaks (DSBs), among the most lethal forms of DNA damage. HR is a high-fidelity repair process that utilizes a homologous DNA sequence as a template to guide the accurate repair of the break. This process is crucial for maintaining genomic stability and preventing mutations that could lead to cancer [4].

PARP’s role in single-strand break (SSB) repair: PARP plays a pivotal role in the repair of SSBs, which are the most common form of DNA damage. PARP detects SSBs and catalyses the addition of poly(adenosine diphosphate ribose) (PAR) chains to itself and other proteins, known as PARylation. This modification recruits and activates various DNA repair proteins at the site of damage [5].

Synthetic Lethality

Concept of synthetic lethality in BRCA-mutated cancers: Synthetic lethality refers to the simultaneous impairment of two genes that leads to cell death, whereas the impairment of either gene alone is non-lethal [6]. In BRCA-mutated cancers, synthetic lethality has been a groundbreaking discovery, particularly in understanding how these tumours can be targeted therapeutically [7]. BRCA1 and BRCA2 are tumour suppressor genes that play a critical role in the repair of double-strand DNA breaks through HR. The HR pathway is compromised when either of these genes is mutated, as commonly observed in certain hereditary breast and ovarian cancers. Despite this defect, cancer cells can survive by relying on alternative DNA repair mechanisms, such as the base excision repair (BER) pathway, to fix SSBs [8].

How PARP inhibitors exploit this mechanism: PARP is an enzyme that plays a key role in the BER pathway, particularly in the repair of SSBs. By blocking the activity of PARP, PARP inhibitors lead to the accumulation of unrepaired SSBs, which eventually convert into DSBs. In cells with functional BRCA1 or BRCA2, the HR pathway would typically repair these breaks. However, in BRCA-mutated cancer cells, the lack of a functional HR pathway means these DSBs cannot be efficiently repaired, leading to genomic instability and cell death [9]. This is where synthetic lethality comes into play: the combination of a BRCA mutation (which impairs HR) and PARP inhibition (which blocks the alternative repair pathway) results in the accumulation of lethal DNA damage, selectively killing cancer cells while sparing normal cells that have a functional HR pathway [10].

Types of PARP inhibitors

Overview of the Most Common PARP Inhibitors

PARP inhibitors represent a groundbreaking class of targeted therapy, especially in the context of advanced ovarian cancer. The most commonly utilized PARP inhibitors include olaparib, niraparib, rucaparib, and talazoparib. Each of these agents has been designed to exploit the concept of synthetic lethality in tumours with deficiencies in HRR, particularly those with BRCA1/2 mutations [11].

Olaparib: The first PARP inhibitor to gain approval, olaparib, has been extensively studied and is approved for use as maintenance therapy in patients with recurrent ovarian cancer, regardless of BRCA mutation status. It is also used in patients with deleterious or suspected deleterious germline or somatic BRCA-mutated advanced ovarian cancer who have been treated with three or more prior lines of chemotherapy [12].

Niraparib: This is another widely used PARP inhibitor, notable for its approval as maintenance therapy in patients with recurrent epithelial ovarian, fallopian tube, or primary peritoneal cancer, regardless of their BRCA mutation status. Niraparib is distinctive due to its efficacy in both BRCA-mutated and BRCA-wild-type ovarian cancers, offering broader applicability in the patient population [13].

Rucaparib: Rucaparib is indicated for the treatment of patients with deleterious BRCA mutations (germline and/or somatic) associated with advanced ovarian cancer who have been treated with two or more chemotherapies. It is also approved as a maintenance treatment in patients with recurrent disease. Rucaparib's ability to target BRCA-mutant and HRD-positive tumours broadens its utility [14].

Talazoparib: This PARP inhibitor is recognized for its potent PARP trapping ability, which enhances its cytotoxicity. Talazoparib is approved for the treatment of germline BRCA-mutated, human epidermal growth factor receptor 2 (HER2)-negative breast cancer, with ongoing studies exploring its efficacy in ovarian cancer. Its unique mechanism may provide advantages in certain subsets of patients, particularly those with specific genetic profiles [15].

Differences in Mechanisms and Clinical Applications

While the primary mechanism of PARP inhibitors involves the inhibition of the PARP enzyme, leading to the accumulation of DNA damage and cell death in HRR-deficient cells, each PARP inhibitor has nuances in its action that can influence its clinical application.

Mechanistic differences: Among the PARP inhibitors, talazoparib is noted for its potent PARP trapping ability, meaning it can more effectively trap PARP-DNA complexes, leading to heightened cytotoxicity compared to other PARP inhibitors. Olaparib, niraparib, and rucaparib also exhibit PARP trapping to varying extents. Niraparib, for instance, is particularly effective across a broader range of genetic backgrounds, including those without BRCA mutations, possibly due to its ability to inhibit PARP-1 and PARP-2 effectively [16].

Clinical applications: The differences in the mechanisms of PARP inhibitors are reflected in their clinical use. Olaparib has been primarily utilized in BRCA-mutant ovarian cancers, though its indications have expanded over time. Niraparib’s efficacy in non-BRCA-mutant patients has made it a more versatile option for maintenance therapy. Rucaparib’s dual utility in both the treatment and maintenance of BRCA-mutant and HRD-positive ovarian cancer emphasizes its broader role in therapy. Talazoparib, though primarily used in BRCA, may offer benefits in ovarian cancer due to its potent PARP-trapping capability, making it a potential option for future clinical use [11].

Clinical trials and efficacy of PARP inhibitors in advanced ovarian cancer

Monotherapy Trials

Monotherapy trials evaluating the efficacy and safety of PARP inhibitors for advanced ovarian cancer showed several key studies demonstrating their effectiveness and safety. One of them is the SOLO-1 trial, which compared olaparib in patients with BRCA-mutated advanced ovarian cancer who observed significant progression-free survival benefits compared to placebo [17]. On the other hand, there was the NOVA trial wherein niraparib was used on both BRCA mutated and non-affected cases so that it can be understood why this particular drug enjoys its fruitful timespan beyond the different types of surgery modalities, including SARATINI I/II and GOG-213 studies; patients have been shown to live longer overall by some measure. Additionally, the ARIEL3 trial emphasized rucaparib’s ability to support the elongation of disease-free periods among those with BRCA or HRD-positive tumours. All these findings talk about how much more one can gain from taking PARP inhibitors alone than when used alongside any other medication, especially when dealing with platinum-based chemotherapy afterwards, which often leads to remission at least once during treatment regime periods [18].

Combination Therapies

In advanced ovarian cancer treatment, combination therapies featuring PARP inhibitors are seen as a promising strategy to boost therapeutic effect and sidestep resistance. Olaparib, niraparib, and rucaparib, which belong to the class of PARP inhibitors, have increasingly been used alongside other agents like chemotherapy, immune checkpoint inhibitors, and targeted therapies to take advantage of their synergetic effects [19]. The aim of combining PARP inhibitors with chemotherapeutic agents is to amplify DNA injury and promote cancer cell apoptosis in a manner that involves more than one mechanism. Furthermore, the addition of immune checkpoint inhibitors combined with PARP inhibitors enhances anti-tumour immunity, thereby improving clinical outcomes. This strategy seeks not only to target DNA repair pathways but also to work on the tumour microenvironment as well as immune evasion, hence offering a holistic treatment option for people suffering from advanced ovarian cancer [20].

Maintenance Therapy

**Figure 1:**
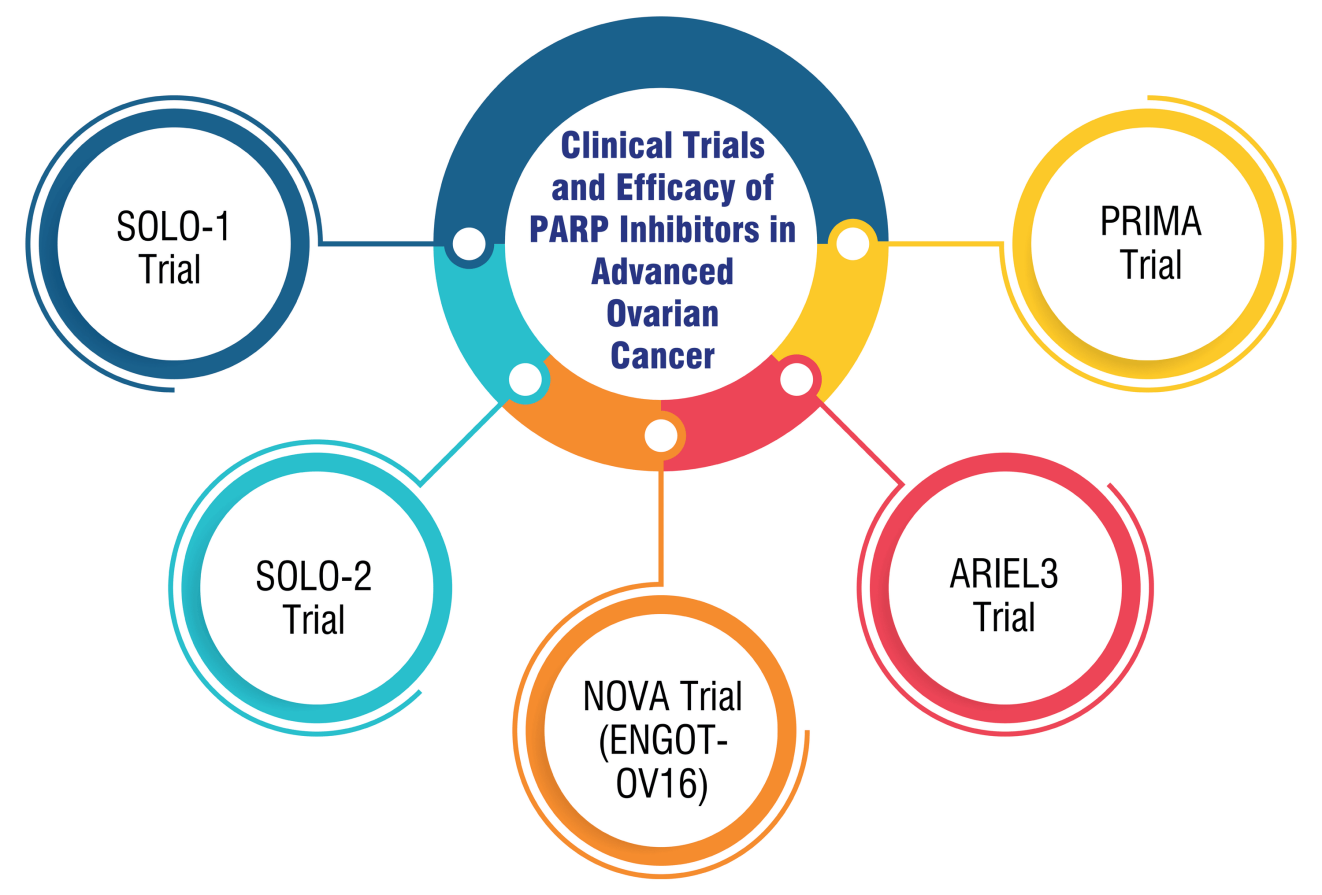
Shows key clinical trials of PARP Image credits: Dr. Arpita Jaiswal PARP: Poly(adenosine diphosphate ribose) polymerase; SOLO-1 trial: Study of olaparib in patients with breast cancer (BRCA) mutations in advanced ovarian cancer; SOLO-2 trial: Study of olaparib in patients with BRCA mutations in recurrent ovarian cancer; PRIMA trial: Phase 3 randomized study of niraparib in patients with advanced ovarian cancer; ARIEL3 trial: A study evaluating rucaparib in patients with relapsed, platinum-sensitive ovarian cancer; NOVA trial: Phase 3 trial of niraparib maintenance treatment in patients with platinum-sensitive, recurrent ovarian cancer; ENGOT: European network of gynaecological oncological trial groups

Maintenance therapy using PARP inhibitors is increasingly being recognized as a paradigm shift in the treatment of patients with advanced ovarian cancer, particularly those harbouring BRCA1/2 mutations or HRD. The principle of action of these compounds, like olaparib, niraparib, and rucaparib, is based on exploiting pre-existing DNA repair defects in tumour cells that ultimately lead to their death due to the inability to restore themselves anymore [21]. For instance, by targeting residual tumour cells after first-line platinum-based chemotherapy, PARP inhibitor maintenance treatment aims to prolong progression-free survival. Consequently, this approach helps control disease progression in a way that maintains low tumour volume, hence improving survival rates. Several clinical trials and observational studies have shown that the use of PARP inhibitors significantly decreases the risk of recurrence among patients with advanced ovarian cancer, making it one of the most important parts of maintenance therapy [22]. Figure 1 shows key clinical trials of PARP inhibitors.

Biomarkers and patient selection

BRCA Mutation Status

The role of BRCA mutation status in advanced ovarian cancer is obvious. Two of these genes, BRCA1 and BRCA2, are involved in the HRR of DSBs. When mutations occur in these genes, it leads to an inability of the cells to repair themselves, which then results in alterations in their DNA, leading to unstable genomes prone to therapy resistance and further disruption of DNA repair pathways. In this way, they utilize PARP inhibitors, which inhibit the PARP enzyme responsible for repairing SSBs in DNA. Therefore, after using this method on BRCA-mutant ovarian cancer cells, cessation occurs when unrepaired pieces accumulate due to a lack of reparation, thus destroying them completely. Patients with BRCA mutations tend to respond well to PARP inhibitors; thus, understanding their mutation statuses becomes paramount for personalizing therapy directed at enhancing performance among people suffering from advanced ovarian tumours [23].

HRD

HRD is important in cases of treatment for advanced cancers, especially, in this case, advanced ovarian cancer, due to the activity of PARP inhibitors. The term HRD is defined as the reduced ability of an organism to repair damaged DNA through a specific mechanism called DSBs. The genesis of HRD in ovarian cancer cells results from mutations in important genes, primarily BRCA1 or BRCA2. This leads to the inability of these cells to accurately repair their own damaged DNA using correct techniques. The following inhibitors, such as olaparib, rucaparib, and niraparib, take advantage of such shortcomings by interfering with their polymerase activity, thus preventing effective repair when they undergo similar injuries; they will eventually die off because there is no more protein synthesis occurring inside them. By targeting specifically tumours that are positive for HRD, this synthetic lethality strategy results in better treatment responses and longer progression-free survival times among patients suffering from late-stage ovarian carcinomas. Therefore, by using genetic tests to determine whether one has HRD, doctors can prescribe PARP inhibitors more precisely to maximize their benefits while providing personalized therapy amidst efforts to manage these types of malignancies [22].

Resistance to PARP inhibitors

Mechanisms of Resistance

There are several mechanisms through which resistance to PARP inhibitors, used to treat advanced ovarian cancer, can develop. The main resistance mechanism is restoring HRR capability, typically through secondary mutations in BRCA1/2 genes, that allow tumour cells to overcome the inhibition of DNA repair mediated by PARP. Moreover, the inefficacy of inhibitors may occur due to modifications in PARP, such as increased expression or mutations, which alter its action. Resistance is also contributed to by the upregulation of alternative DNA repair pathways, like non-homologous end joining (NHEJ), and overexpression of drug efflux pumps. Tumour microenvironment factors, including epigenetic modifications, also modify the response to PARP inhibitors. This understanding of resistance would help develop ways to overcome it, to enhance treatment outcomes for advanced ovarian cancer [24].

Overcoming Resistance

Overcoming the resistance to PARP inhibitors remains one of the most difficult tasks in curing advanced ovarian cancer. Although they are effective in targeting cancer cells with defective DNA repair mechanisms, several other factors can lead to resistance, such as increased drug efflux, restoration of HRR, or changes in drug targets. Additionally, BRCA1/2 secondary mutations, as well as mutations in other DNA repair genes, may decrease their efficacy. Ongoing research is concentrated on combination therapies that involve new compounds targeting the pathways of response to DNA damage, alongside strategies to overcome resistance mechanisms. Improved patient outcomes and wider accessibility of PARP inhibitors can be achieved through personalized treatment approaches utilizing genetic profiling and continuous observation of treatment reactions [25].

Safety and toxicity profile

Common Adverse Effects

Whereas they are useful in the management of advanced ovarian cancer, PARP inhibitors have several common side effects. Some of these side effects are haematological, such as anaemia and neutropenia. These can increase the risk of infections, bleeding, or even fatigue. The risk of infections, bleeding, and fatigue are some of the haematological complications associated with them. They also present with gastrointestinal problems, such as nausea, vomiting, or diarrhoea. Fatigue is frequent among patients, which might affect their general well-being. Elevated liver enzymes may be noticed on rare occasions, indicating impending liver impairment. More serious complications, though rare, may arise from PARP inhibitors, such as myelodysplastic syndromes or acute myeloid leukaemia, necessitating proper monitoring and management throughout the treatment process [26].

Long-Term Safety Concerns

When it comes to patients with advanced ovarian cancer, long-term safety concerns surrounding the use of PARP inhibitors are very important in terms of treatment planning and management. While these drugs have shown efficacy in prolonging progression-free survival rates and improving overall outcomes, they also come with some possible long-term side effects. These can include extreme malignancies that might develop after years, such as acute myeloid leukaemia or myelodysplastic syndromes. In addition, another concern is that if you continue taking them for a long time, you may eventually suffer from cumulative toxicities, such as haematological abnormalities like anaemia, thrombocytopenia, or neutropenia, as well as impacts on kidney and liver functions. Continuous monitoring, as well as further research, is important to fully understand the long-term safety profile of PARP inhibitors and, therefore, to develop improved management strategies for patients undergoing extended therapy periods [27].

Comparative Toxicity

Olaparib, niraparib, and rucaparib are examples of PARP inhibitors used for advanced ovarian cancer treatment. These medications show promising activity in advanced cases, but their potential side effects must be considered. The drugs mainly act by targeting BRCA1/2-mutant cancer cells and other HRDs, leading to synthetic lethality. Nonetheless, they may produce varying degrees of toxicity. Common toxicities include hematologic complications, such as anaemia, thrombocytopenia, and neutropenia, which may require dosage adjustments or discontinuation of treatment.

Furthermore, nausea and vomiting are common, along with gastrointestinal issues. Additionally, patients may experience fatigue and, occasionally, risk for secondary malignancies, such as myelodysplastic syndrome or acute myeloid leukaemia. Comparative toxicity analysis indicates that, although PARP inhibitors generally have a manageable safety profile, the side effects vary by agent, making them suitable for personalized treatment plans that require close monitoring to improve patient outcomes [11].

Future directions and emerging research

In recent years, both the American Society of Clinical Oncology (ASCO) and the European Society for Medical Oncology (ESMO) have emphasized the importance of personalized medicine in the treatment of advanced ovarian cancer, particularly concerning the use of PARP inhibitors. ASCO has highlighted the need for further research to identify biomarkers that predict response to PARP inhibitors, advocating for trials that explore combination therapies to enhance efficacy and overcome resistance. ESMO has similarly focused on optimizing patient selection and tailoring treatments based on genetic profiling, with an emphasis on integrating PARP inhibitors into broader therapeutic strategies. Future directions in the use of PARP inhibitors for advanced ovarian cancer are poised to expand beyond their current indications and combinations. Ongoing research is exploring the potential of these inhibitors in earlier stages of the disease, aiming to enhance their efficacy and reduce recurrence rates. There is also increasing interest in optimizing treatment regimens by combining PARP inhibitors with other targeted therapies, immunotherapies, or novel agents to overcome resistance and improve patient outcomes. Personalized medicine approaches, including genetic and molecular profiling, are anticipated to refine patient selection and tailor treatments more precisely. Additionally, studies are investigating the long-term effects and safety profiles of PARP inhibitors and their impact on quality of life. As research progresses, these advancements could lead to more effective and individualized treatment strategies, ultimately enhancing survival and therapeutic success for patients with advanced ovarian cancer.

## Conclusions

In conclusion, PARP inhibitors represent a significant advancement in treating advanced ovarian cancer, offering an encouraging medical alternative, especially for BRCA1/2 mutants and patients with HR defects. The statement underscores their role in both maintenance and treatment situations, highlighting how effective they can be at improving not only progression-free survival but also response rates, generally speaking. However, despite these developments, some challenges still exist, such as dealing with resistance mechanisms and determining the best groups of individuals to target with this treatment modality. As such, future inquiries should aim to overcome these bottlenecks and explore combination strategies to enhance therapy outcomes. All in all, PARP inhibitors have revolutionized the management of advanced ovarian cancer, offering hope for better patient care and improved quality of life.
